# High Salivary 3-Nitrotyrosine Levels in Periodontitis

**DOI:** 10.3390/jcm14196785

**Published:** 2025-09-25

**Authors:** Leonardo Lorente, Esther Hernández Marrero, Pedro Abreu González, Angel Daniel Lorente Martín, Agustín F. González-Rivero, María José Marrero González, Carmen Hernández Marrero, Olga Hernández Marrero, Alejandro Jiménez, Cándido Manuel Hernández Padilla

**Affiliations:** 1 Intensive Care Unit, Hospital Universitario de Canarias, Ofra s/n, 38320 San Cristóbal de La Laguna, Tenerife, Spain; 2 Clínica Dental Cándido, Plaza San Cristóbal 35, 38204 San Cristóbal de La Laguna, Tenerife, Spain; esther@clinicadentalcandido.com (E.H.M.); mariajose@clinicadentalcandido.com (M.J.M.G.); carmen@clinicadentalcandido.com (C.H.M.); olga@clinicadentalcandido.com (O.H.M.); candido@clinicadentalcandido.com (C.M.H.P.); 3 Unit of Physiology, Department of Basic Medical Sciences, Faculty of Medicine, University of La Laguna, Ofra s/n, 38320 San Cristóbal de La Laguna, Tenerife, Spain; pabreu@ull.edu.es; 4 Department of Odontology, Faculty of Medicine, CEU San Pablo University, Avenida Montepríncipe s/n, 28660 Boadilla del Monte, Madrid, Spain; daniellorentemartin@gmail.com; 5 Laboratory Department, Hospital Universitario de Canarias, Ofra s/n, 38320 San Cristóbal de La Laguna, Tenerife, Spain; afgonriv@gmail.com; 6 Research Unit, Hospital Universitario de Canarias, Ofra s/n, 38320 San Cristóbal de La Laguna, Tenerife, Spain; ajimenezsosa@gmail.com

**Keywords:** 3-nitrotyrosine, oxidation, nitrosidation, salivary, periodontitis

## Abstract

**Background:** Tyrosine, a non-essential amino acid involved in protein biosynthesis, can undergo oxidative modification upon exposure to reactive species like the peroxynitrite radical (ONOO^−^), resulting in the formation of 3-nitrotyrosine (3-NT). High concentrations of 3-NT have been found in the periodontal tissues of rats with periodontitis and in one study involving twelve patients with periodontitis; further, a correlation between 3-NT concentrations in periodontal tissues and clinical indices of periodontitis was found in 24 patients with periodontitis. The objectives of our study were to compare salivary 3-NT concentrations in subjects with and without periodontitis, to determine whether an association exists between salivary 3-NT concentrations and periodontitis, and to evaluate the potential of salivary 3-NT concentrations for the diagnosis of periodontitis. **Material and Methods:** This prospective study involved the measurement of salivary 3-NT concentrations in individuals diagnosed with periodontitis—defined by the presence of periodontal tissue loss—and in individuals without periodontitis, characterized by either periodontal health or localized gingivitis affecting fewer than 30% of sites. A total of 66 participants without periodontitis and 60 with periodontitis were included. To identify variables independently linked to periodontitis, multivariate logistic regression was applied. Additionally, a receiver operating characteristic (ROC) analysis was carried out to assess the ability of salivary 3-NT levels to discriminate between the two groups. **Results:** Multivariate logistic regression analysis showed that the variables independently associated with periodontitis were salivary 3-NT concentrations > 4.25 ng/mL (OR = 3.22; 95% CI = 1.180–8.789; *p* value = 0.02), age (years) (OR = 1.12; 95% CI = 1.064–1.168; *p* value < 0.001), and being a never smoker (OR = 0.36; 95% CI = 0.129–0.989; *p* value = 0.048). The area under the curve (AUC) of salivary 3-NT concentrations for the diagnosis of periodontitis was 62% (95% CI = 53–70%; *p* value = 0.02). **Conclusions:** Our findings showed that salivary 3-NT concentrations were higher in subjects with periodontitis than in those without, that there is an association between high salivary 3-NT concentrations and periodontitis, and that salivary 3-NT concentrations may be useful for diagnosing periodontitis.

## 1. Introduction

Periodontitis is a highly prevalent chronic condition involving sustained inflammation of the periodontal tissues, which may result in the destruction of bone and eventual tooth loss. The associated burden on healthcare systems is substantial [1].

Several pathophysiological pathways are activated during periodontitis, including inflammation [2,3,4,5], programmed cell death [6,7], oxidation [8,9,10] and nitrosation [11,12,13,14,15].

Tyrosine is a non-essential amino acid that plays a central role in cellular protein synthesis. The high reactive anion species of superoxide (O_2_^●−^) and nitric oxide (NO) form the free radical peroxynitrite (ONOO^−^). When tyrosine is attacked by ONOO^−^, it forms the pro-oxidant compound 3-nitrotyrosine (3-NT) [11,12,13,14,15].

Saliva has recently gained attention in clinical diagnostics due to its low cost, stability, and noninvasive, painless collection—even in pediatric populations [16]. Various methods exist for saliva collection, including unstimulated and stimulated techniques [17,18,19,20]. Although saliva is composed primarily of water, it also contains electrolytes, amino acids, proteins, lipids, hormones, vitamins, antioxidants, and oxidation products of biomolecules. These compounds can pass from blood into saliva via passive mechanisms (simple diffusion, ultrafiltration, facilitated diffusion), active transport, or through damaged cell membranes [21]. The composition of saliva reflects dynamic physiological changes in the body, making it a promising tool for identifying clinically valuable biomarkers. The analysis of salivary reactive species and antioxidants may be useful as diagnostic, prognostic, or therapeutic markers—not only for oral diseases but also for systemic conditions [22]. Nitrosative stress is characterized as the imbalance between reactive nitrogen species and antioxidants, in favor of pro-oxidant activity. Salivary biomarkers of nitrosative stress may prove useful in dental practice [23].

Higher concentrations of 3-NT have been found in the periodontal tissues of rats with periodontitis compared to those without [24,25,26]. In addition, a study involving twelve patients with periodontitis reported higher 3-NT levels in periodontal tissues than in healthy controls [27]. Another study found a correlation between 3-NT concentrations in gingivomucosal biopsy tissues and clinical indices of periodontitis in twenty-four patients [28]. The objectives of our study were to compare salivary 3-NT concentrations in subjects with and without periodontitis, to determine whether an association exists between salivary 3-NT levels and periodontitis, and to evaluate the potential utility of salivary 3-NT concentrations for an optimum diagnosis of periodontitis.

## 2. Methods

### 2.1. Design and Subjects

This prospective observational study was initiated after approval by the Clinical Research Ethics Committee of the Hospital Universitario de Canarias (CHUC_2023_138; 30 November 2023). Written informed consent was obtained from each participant prior to inclusion in the study.

Subjects with periodontitis (defined by clinical evidence of periodontal tissue loss) and subjects without periodontitis (defined by evidence of localized gingivitis in <30% of sites or by periodontal health) were included. Internationally accepted diagnostic and classification criteria were used to identify and categorize periodontitis and its severity [29]. Exclusion criteria included lactating females and individuals younger than 18 years. Subject recruitment was conducted by Clínica Dental Cándido (La Laguna, Tenerife, Canary Islands, Spain). The first subject was enrolled in January 2024.

### 2.2. Definitions

Clinical periodontal health was defined as the absence of bleeding on probing or bleeding present in <10% of sites, with no evidence of clinical interproximal attachment loss or bone loss.

Localized gingivitis was defined as bleeding present in 10–30% of sites, without clinical interproximal attachment loss or bone loss.

Periodontitis was defined as the presence of clinical interproximal attachment loss or bone loss. Periodontitis severity was classified based on the following criteria: (1) Clinical interproximal attachment loss: <3 mm for stage I, 3 to 4 mm for stage II, ≥5 mm for stages III or IV. (2) Radiographic bone loss: coronal third < 15% for stage I, coronal third 15–33% for stage II, middle or apical third for stages III or IV. (3) Loss of teeth: none for stage I, 1 to 4 for stage II, ≥5 teeth for stage III.

Periodontal probing was performed using PCP-12 probes (Hu-Friedy, Chicago, IL, USA). All teeth in each subject were examined at six sites per tooth: mesial, central, and distal on both the lingual (internal) and buccal (external) surfaces.

### 2.3. Variables Recorded

Age, sex, diabetes mellitus, cardiovascular disease, arterial hypertension, hypercholesterolemia, body mass index (BMI, kg/m^2^), obesity (BMI ≥ 30 kg/m^2^), oral cancer, rheumatoid arthritis, systemic lupus erythematosus, methotrexate at dosage for rheumatoid arthritis, radiotherapy, immunosuppressive therapy, dental hygiene, and consumption of tobacco, drugs, tea, alcohol and coffee were recorded for all subjects.

### 2.4. Salivary Samples

Whole unstimulated saliva samples were collected using the technique described by Navazesh [30]. Samples were collected in the morning (between 8:00 and 10:00 a.m.) to minimize potential alterations in salivary biomarker concentrations due to circadian rhythm. Participants were instructed not to drink, smoke, or brush their teeth within 2 h prior to sample collection. Before collection, each participant rinsed their mouth three times with 10 mL of deionized water. Then, they were seated comfortably with their eyes open, avoiding orofacial movements, and with their heads tilted slightly forward for 30 min. During this time, they refrained from swallowing, allowing saliva to accumulate in the mouth before expectorating it into a sterile container. Saliva samples were immediately centrifuged at 3000 rpm for 10 min at 24 °C to remove cells and debris. The supernatant was pipetted into Eppendorf tubes and stored at −80 °C until analysis.

Some of the subjects had also enrolled in prior publications from the same research team. However, those studies focused on salivary levels of nitrites [31], uric acid [32] and malondialdehyde [33]. However, the objective of the present study was to determine salivary 3-nitrotyrosine

### 2.5. Salivary 3-NT Concentrations Analysis

Salivary concentrations of 3-NT were measured using a solid-phase competitive Enzyme Linked Immunosorbent Assay (Invitrogen, Thermo Fisher Scientific Inc., Whaltam, MA, USA), according to the manufacturer’s instructions. Briefly, 50 μL of standards, 50 μL of undiluted saliva samples, and 50 μL of biotinylated detection antibody working solution were added to the appropriate wells and incubated for 45 min at 37 °C. After washing the wells with wash buffer, 100 μL of horseradish peroxidase (HRP) conjugate working solution was added to each well and incubated for 30 min at 37 °C. After a second wash, 90 μL of substrate reagent (3,3′,5,5′-tetramethylbenzidine [TMB]) was added and incubated for 15 min at 37 °C, avoiding direct light. The reaction was stopped by adding 50 μL of stop solution (1 M H_2_SO_4_) to each well, resulting in a yellow color. A calibration curve ranging from 0 to 100 ng/mL was used. Absorbance values of samples and standards were read at 450 nm using a microplate spectrophotometer (Spectra MAX-190, Molecular Devices, Sunnyvale, CA, USA). A four-parameter logistic (4PL) curve-fitting algorithm was used to generate the standard curve, with correlation coefficients (r^2^) ranging from 0.9932 to 0.9967 across different kits. The lower limit of detection was 0.64 ng/mL. Intra-assay and inter-assay coefficients of variation were 5.21% and 5.43%, respectively.

### 2.6. Statistical Methods

The Mann–Whitney U test and the chi-square test were used to compare subjects with and without periodontitis for continuous variables (reported as median and interquartile range [IQR]) and categorical variables (reported as number and percentage), respectively. The association between salivary 3-NT concentrations and periodontal stage was evaluated using Spearman’s rho correlation coefficient.

A multivariate logistic regression analysis was performed to identify variables independently associated with periodontitis. Variables included in the regression were those with a *p* value ≤ 0.05 in the univariate comparisons between groups and with an adequate number of events in each category of the independent variables. These variables included salivary 3-NT concentrations, smoking status, age, and arterial hypertension.

Additionally, a propensity score analysis was conducted to account for baseline differences between the two groups. Variables included in the propensity score model were those that showed a *p* value < 0.05 in group comparisons and had sufficient numbers of events. Age, arterial hypertension, and smoking status were used as independent variables, and salivary 3-NT concentration was set as the dependent variable. The resulting propensity scores and salivary 3-NT concentrations were then included in a final multivariate logistic regression analysis to predict periodontitis status. This approach helped to balance the distribution of key covariates between subjects with and without periodontitis.

A receiver operating characteristic (ROC) curve analysis was performed to evaluate the diagnostic capability of salivary 3-NT concentrations for periodontitis. The area under the curve (AUC) and its 95% confidence interval (CI) were reported. Specificity, sensitivity, positive and negative predictive values, and positive and negative likelihood ratios for a salivary 3-NT cut-off > 4.25 ng/mL were also reported with corresponding 95% CIs. This cut-off value was selected based on the Youden J index [34].

The association between salivary 3-NT concentrations and categorical variables was assessed using the Mann–Whitney U test, while correlations with continuous variables were analyzed using Spearman’s rho correlation coefficient.

All statistical analyses were performed using SPSS version 17.0 (SPSS Inc., Chicago, IL, USA), LogXact-4 for Windows version 4.1 (Cytel Software Corporation, Cambridge, MA, USA), and MedCalc Statistical Software version 22.016 (MedCalc Software Ltd., Ostend, Belgium).

## 3. Results

A total of 66 subjects without periodontitis and 60 subjects with periodontitis were included in the study. The number of subjects and corresponding salivary 3-NT concentrations across periodontal stages are shown in Table 1. A positive correlation was found between salivary 3-NT concentrations and periodontal stage (Spearman’s rho = 0.22; *p* value = 0.02).

Subjects with periodontitis had higher salivary 3-NT concentrations compared to those without periodontitis (*p* value = 0.02), were older (*p* value < 0.001), and had higher rates of never smoking history (*p* value < 0.001), arterial hypertension (*p* value = 0.001), diabetes mellitus (*p* value = 0.02), and cardiovascular disease (*p* value = 0.049). No statistically significant differences were observed between subjects with and without periodontitis regarding sex, hypercholesterolemia, rheumatoid arthritis, methotrexate use at dosages for rheumatoid arthritis, immunosuppressive therapy, radiotherapy, coffee, alcohol, tea consumption, body mass index, or obesity (Table 2). None of the subjects included in the study (with or without periodontitis) had oral cancer or systemic lupus erythematosus, nor did they report drug use.

Multiple logistic regression analysis showed that the variables associated independently with periodontitis were salivary 3-NT concentrations > 4.25 ng/mL (OR = 3.22; 95% CI = 1.180–8.789; *p* = 0.02), age (years) (OR = 1.12; 95% CI = 1.064–1.168; *p* < 0.001), and never smoker (OR = 0.36; 95% CI = 0.129–0.989; *p* = 0.048) (Table 3).

Propensity analysis showed similar results. Salivary 3-NT concentrations > 4.25 ng/mL remained independently associated with periodontitis (OR = 2.67; 95% CI = 1.13–6.33; *p* = 0.03).

No significant associations were found between salivary 3-NT levels and sex (*p* = 0.85), age (rho = 0.02; *p* = 0.79), arterial hypertension (*p* = 0.23), cardiovascular disease (*p* = 0.90), hypercholesterolemia (*p* = 0.86), diabetes mellitus (*p* = 0.99), rheumatoid arthritis (*p* = 0.23), metrotexate (*p* = 0.27), immunosupressive therapy (*p* = 0.08), radiotherapy (*p* = 0.24), body mass index (rho = −0.004; *p* = 0.97), obesity (*p* = 0.22), never smoker (*p* = 0.82), coffee (*p* = 0.67), tea (*p* = 0.54) and alcohol (*p* = 0.25).

**Figure 1:**
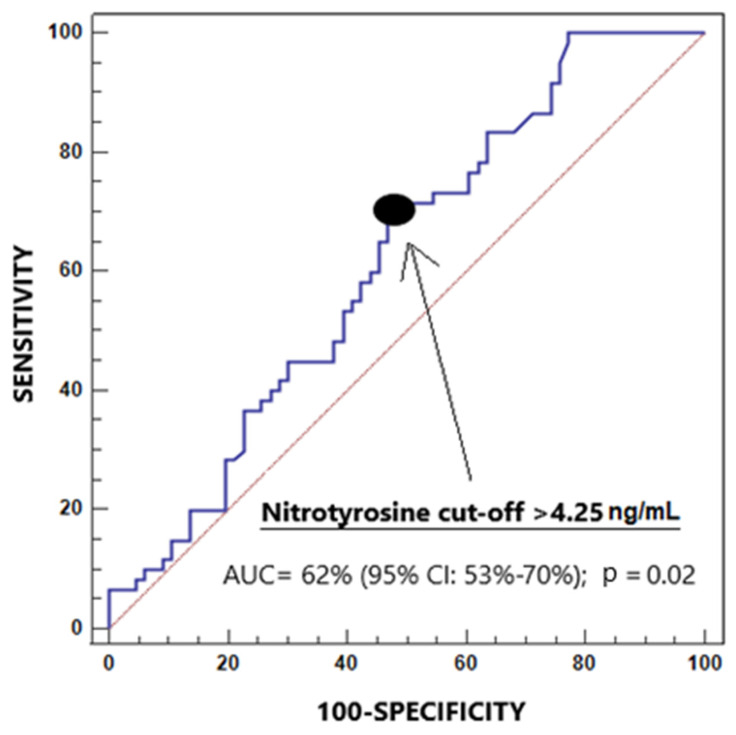
Receiver operating characteristic analysis to determine the capability of salivary 3-nitrotyrosine concentrations for periodontitis diagnosis.

AUC of salivary 3-NT levels for the diagnosis of periodontitis was 62% (95% CI = 53–70%; *p* = 0.02) (Figure 1). The cut-off point > 4.25 ng/mL of salivary 3-NT levels for periodontitis diagnosis had positive likelihood ratio 1.5 (1.1–2.0), negative likelihood ratio 0.6 (0.4–0.9), sensitivity 72% (59–83%), positive predictive value 57% (50–64%), negative predictive value of 67% (56–76%) and specificity 52% (39–64%).

## 4. Discussion

We found that salivary 3-NT levels were significantly higher in periodontitis patients than in controls, that elevated concentrations are linked to the presence of the disease, and that 3-NT may support diagnostic efforts in periodontitis.

Our findings are consistent with previous findings of increased 3-NT concentrations in periodontal tissues of rats with periodontitis compared to controls [24,25,26], and in periodontal tissues of 12 patients with periodontitis compared to control subjects [27], and with the correlation between 3-NT concentrations in tissues of gingivomucosal biopsies and the periodontitis clinical indices in 24 patients with periodontitis [28].

We also found a correlation between salivary 3-NT concentrations and periodontitis severity (rho = 0.22; *p* = 0.02); however, it was relatively low and lower than the correlation found in the study by Pârvu et al. between gingivomucosal 3-NT concentrations and the periodontitis clinical indices (*r* = 0.65–0.94) [28].

A higher correlation has been found between salivary levels of malondialdehyde (a biomarker of lipid peroxidation) and periodontitis severity, according to the results from a study by Nanakaly et al. (*r* = 0.56; *p* = 0.001) [35] and another study by our group (rho = 0.40; *p* < 0.001) [33].

Different risk factors of periodontitis have been reported, such as systemic lupus erythematosus [36], rheumatoid arthritis [37], sex [38], age [39], diabetes mellitus [40], arterial hypertension [41], obesity [42], consumption of drugs, tobacco, tea, alcohol or coffee, dental hygiene, oral cancer and immunosuppression [43]. We also found that age [39] and tobacco [43] were independently associated with periodontitis. We did not find other variables independently associated with periodontitis in our study.

From a therapeutic perspective, the results of some studies suggest the possibility to modulate 3-NT concentrations in gingivomucosal tissue [28,44,45]. In animal models of rats with periodontitis, we found that the oral administration of some agents (resveratrol or GW0742) reduced 3-NT concentrations in gingivomucosal tissue and improved clinical attachment [44,45]. In a randomized clinical trial published by Pârvu et al. in 2013 [28], the effectiveness of subantimicrobial-dose doxycycline (SDD) administered orally, as an adjunct to scaling and root planing (SRP) treatment was evaluated against the nitrosative stress of moderate to advanced chronic periodontitis. The effect of SDD on clinical attachment state was analyzed in all patients included in the study (n = 174) and SDD was associated with better clinical attachment state. The effect of SDD on 3-NT concentrations in gingivomucosal tissue was analyzed in 24 of those patients and SDD was associated with lower 3-NT concentrations [28].

Several factors may influence both the clinical identification and severity grading of periodontitis, including patient discomfort during examination, restricted mouth opening, and the probing force applied within the gingival sulcus [2]. Thus, salivary 3-NT concentrations could be used for the clinical diagnosis of periodontitis, the periodontitis severity classification, and to monitor the evolution and response to treatment.

We admit having some limitations in our study. We have not measured concentrations of 3-NT in other samples (gingival crevicular fluid or blood); however, we have only handled saliva samples to be less invasive. The relatively low sample size of our study forced us to include in the group of subjects without periodontitis those with periodontal health and localized gingivitis. Besides, perhaps with a larger sample size we would have found more variables independently associated with periodontitis. In addition, we have not analyzed the effects of diet or socioeconomic status in our findings. The use of some medications, such as ibuprofen [46], could modify the levels of 3-NT; and we did not register the use and dosage of all medications used by the subjects. We have not measured total tyrosine levels to explore whether a ratio between 3-NT and total tyrosine is less/same/more predictive than only 3-NT levels.

The periodontitis subject group presented higher age and a greater prevalence of comorbidities than the group of subjects without periodontitis, which could partly explain the observed differences in salivary 3-NT concentrations between both groups of subjects. Although we adjusted for these variables using multiple logistic regression analysis, we acknowledge that regression alone may not fully address the imbalance between groups, particularly given the observational nature of the study. To address this limitation, we performed an additional analysis using propensity score methods to account for baseline differences between both groups. The variables selected for the propensity score were age, arterial hypertension and smoking status as independent variables and salivary 3-NT concentrations as dependent variable. The propensity scores and salivary 3-NT concentrations variables were then included in a final multiple logistic regression analysis to predict periodontitis status. This approach allows us to better balance the distribution of influential covariates between subject groups (with and without periodontitis). The results of this analysis were consistent with our main findings, further supporting the robustness of the association between periodontitis and salivary 3-NT concentrations.

In addition, we have not found an association between salivary 3-NT levels and sex, age, arterial hypertension, cardiovascular disease, hypercholesterolemia, diabetes mellitus, rheumatoid arthritis, methotrexate, immunosuppressive therapy, radiotherapy, body mass index, obesity, never smoker, coffee, tea and alcohol. It is possible that other variables that were not registered in our study could influence salivary 3-NT levels. However, the finding of the association between salivary 3-NT levels and periodontitis and the absence of the association between salivary 3-NT levels and other variables in our study also support the robustness of the association between periodontitis and salivary 3-NT concentrations.

Periodontitis diagnosis is carried out using clinical and radiological criteria. However, the clinical examination could be influenced by factors such as limited mouth opening, discomforts during the exploration and the pressure applied when the periodontal probe is used [2]. Therefore, the determination of biomarkers could be helpful for the diagnosis of periodontitis or for periodontitis severity estimation [2]. According to the results of our study, the determination of salivary 3-NT concentrations to assess the salivary oxidant status of patients with periodontitis could be proposed. In addition, it could be proposed to promote the administration of antioxidant agents in the form of mouth creams for patients with periodontitis to minimize its severity progression or to improve its periodontal state; logically, that therapeutic approach could be considered after conducting studies in animals and humans.

We think that the novel findings of our study (despite its limitations) about salivary 3-NT concentrations and the findings of previous studies in rat models and in patients with the possibility to modulate 3-NT tissue concentrations could motivate further research into 3-NT in periodontitis.

## 5. Conclusions

This study demonstrated that salivary 3-NT concentrations are increased in patients with periodontitis, show a significant association with disease status, and could serve as a non-invasive biomarker for diagnosis.

## Figures and Tables

**Table 1:** Number of subjects and salivary 3-nitrotyrosine concentrations in each periodontal stage.

	Total (*n* = 126)	3-Nitrotyrosine (ng/mL) Median (p 25–75)
Without Periodontitis—*n* (%)	66 (52.4)	4.20 (2.53–8.95)
Periodontitis stage I—*n* (%)	17 (13.5)	5.68 (2.53–10.43)
Periodontitis stage II—*n* (%)	24 (19.0)	5.88 (3.79–9.48)
Periodontitis stage III—*n* (%)	13 (10.3)	5.85 (3.32–10.90)
Periodontitis stage IV—*n* (%)	6 (4.8)	7.27 (4.38–21.35)

**Table 2:** Comparison between subjects with and without periodontitis.

	Subjects Without Periodontitis (*n* = 66)	Subjects with Periodontitis (*n* = 60)	*p*-Value
Gender female—*n* (%)	47 (71.2)	37 (61.7)	0.26
Age (years)—median (*p* 25–75)	40 (30–48)	60 (51–68)	<0.001
Arterial hypertension—*n* (%)	4 (6.1)	18 (30.0)	0.001
Cardiovascular disease—*n* (%)	0	4 (6.7)	0.049
Hyperocholesterolemia—*n* (%)	2 (3.0)	2 (3.3)	0.99
Diabetes mellitus—*n* (%)	0	5 (8.3)	0.02
Rheumatoid arthritis—*n* (%)	1 (1.5)	4 (6.7)	0.19
Metrotexate for rheumatoid arthritis—*n* (%)	1 (1.5)	0	0.99
Immunosupressive therapy—*n* (%)	1 (1.5)	2 (3.3)	0.61
Radiotherapy—*n* (%)	0	2 (3.3)	0.23
Body mass index (kg/m^2^)—median (*p* 25–75)	24.5 (22.4–26.6)	24.8 (22.6–28.2)	0.37
Obesity—*n* (%)	7 (10.6)	9 (15.0)	0.59
Never smoker—*n* (%)	54 (81.8)	25 (41.7)	<0.001
Coffee—*n* (%)	55 (83.3)	53 (88.3)	0.46
Tea—*n* (%)	9 (13.6)	3 (5.0)	0.13
Alcohol—*n* (%)	25 (37.9)	31 (51.7)	0.15
Salivary 3-NT levels (ng/mL)—median (*p* 25–75)	4.20 (2.53–8.95)	5.78 (3.62–9.69)	0.02
Salivary 3-NT levels > 4.25 ng/mL—*n* (%)	32 (48.5)	43 (71.7)	0.01

3-NT: 3-nitrotyrosine.

**Table 3:** Multiple logistic regression analysis of factors associated with periodontitis.

	Odds Ratio	95% Confidence Interval	*p*-Value
Age (years)	1.12	1.064–1.168	<0.001
Salivary 3-nitrotyrosine levels > 4.25 ng/mL	3.22	1.180–8.789	0.02
Never smoker (yes vs. non)	0.36	0.129–0.989	0.048
Arterial hypertension (yes vs. non)	1.13	0.260–4.865	0.88

## Data Availability

The data that supports the findings of this study are available from the corresponding author upon reasonable request.

## Abbreviations

3-NT: 3-nitrotyrosine
